# Development of a New Index of Balance in Adults with Intellectual and Developmental Disabilities

**DOI:** 10.1371/journal.pone.0096529

**Published:** 2014-05-02

**Authors:** Antonio Cuesta-Vargas, Maria Giné-Garriga

**Affiliations:** 1 Department of Physical Therapy, Universidad de Málaga, Málaga, Spain; 2 School of Clinical Sciences of the Faculty of Health at the Queensland University of Technology, Brisbane, Australia; 3 Department of Physical Activity and Sport Sciences, FPCEE Blanquerna, Universitat Ramon Llull, Barcelona, Spain; 4 Department of Physical Therapy, FCS Blanquerna, Universitat Ramon Llull, Barcelona, Spain; Shanghai Mental Health Center, Shanghai Jiao Tong University School of Medicine, China

## Abstract

**Purposes:**

The first objective was to propose a new model representing the balance level of adults with intellectual and developmental disabilities (IDD) using Principal Components Analysis (PCA); and the second objective was to use the results from the PCA recorded by regression method to construct and validate summative scales of the standardized values of the index, which may be useful to facilitate a balance assessment in adults with IDD.

**Methods:**

A total of 801 individuals with IDD (509 males) mean 33.1±8.5 years old, were recruited from Special Olympic Games in Spain 2009 to 2012. The participants performed the following tests: the *timed-stand test*, the *single leg stance test* with open and closed eyes, the *Functional Reach Test*, the E*xpanded Timed-Get-up-and-Go Test*. Data was analyzed using principal components analysis (PCA) with Oblimin rotation and Kaiser normalization. We examined the construct validity of our proposed two-factor model underlying balance for adults with IDD. The scores from PCA were recorded by regression method and were standardized.

**Results:**

The Component Plot and Rotated Space indicated that a two-factor solution (*Dynamic and Static Balance components*) was optimal. The PCA with direct Oblimin rotation revealed a satisfactory percentage of total variance explained by the two factors: 51.6 and 21.4%, respectively. The median score standardized for component dynamic and static of the balance index for adults with IDD is shown how references values.

**Conclusions:**

Our study may lead to improvements in the understanding and assessment of balance in adults with IDD. First, it confirms that a two-factor model may underlie the balance construct, and second, it provides an index that may be useful for identifying the balance level for adults with IDD.

## Introduction

Whilst no one definition of intellectual and developmental disabilities (IDD) has gained universal acceptance, it is generally accepted that the term IDD encompasses any set of conditions, resulting from genetic, neurological, nutritional, social, traumatic or other factors occurring prior to birth, at birth, or during childhood up to the age of brain maturity, that affect intellectual development [1]. These conditions may occur in conjunction with physical, sensory or psychiatric impairments of varying degree [2].

Increased longevity and more readily available services have led to an increase of older adults with IDD in the developed nations of the world [1]. Locomotor skills in people with IDD are characterized by decreased accuracy, adults with mild or moderate IDD are often found to have sensory integration problems [3] and a sedentary lifestyle [4] [5] [6]. Balance and mobility limitations are common in persons with IDD. Differences in balance and mobility between persons with IDD and controls have mainly been demonstrated by instrumented assessments (e.g. force platforms, posturography and gait analysis), which require sophisticated and expensive equipment such as force plates or a 3D motion analysis system [7]. Most physicians and allied healthcare professionals working with persons with IDD do not have such equipment at their disposal, so they must rely on clinical tests to determine whether mobility is affected.

Individuals that have IDD are particularly at risk of balance and mobility impairment concerning the potential development of deficits in both locomotor skills as in daily functioning [8]. Scientists had paid little attention to adults with IDD in general and to their balance deficiency in particular. It is imperative to gain insight into the severity and prevalence of balance problems in this population, which present a special challenge to the healthcare system. As to date, it remains unclear which specific balance test is feasible and reliable for testing subjects with IDD. While performance-based balance measures are used extensively to assess balance ability and monitor change, existing measures have important limitations in individuals with IDD [9], including limited comprehensiveness in content, ceiling effects, and limited sensitivity to change and responsiveness [10] [11]. These limitations likely contribute to multiple test administration for balance assessment increasing burden [12].

Studying balance in older persons with IDD, which is important for maintaining many activities of daily living, and being able to construct a balance index specifically for adults with IDD might be a valuable tool in classifying population with IDD and assessing their risk of falling. Most balance measures are traditional fixed-form measures, requiring administration of fixed item sets to every person. Designing psychometrically strong fixed form balance measures is challenging due to the large number of items needed to encompass the spectrum of balance ability and components [13].

Accordingly, our study aimed to attain two main objectives: [14] the first one was to propose a new model representing the balance level of adults with IDD using Principal Components Analysis (PCA); [15] and the second objective was to use the results from the PCA recorded by regression method to construct and validate summative scales of the standardized values of the index, which may be useful to facilitate a balance assessment in adults with IDD.

## Methods

### Participants

To carry out this study ethical principles were taken into account. The institutional review board at the University of Malaga approved the procedures used in this study, and all of the participants and their parents or guardians gave their written informed consent before participation There were defined and established by the Declaration of Helsinki and according to the standards of Good Clinical Practice (GCP). Before starting the investigation we have guaranteed to the participants the protection of confidential information obtained by them [Law 15/1999 Protection of Personal Data]. In all cases we ensure the anonymity of participants. Also be detailed at all times that participation in the study is voluntary and they have to firm written informed consent.

A total of 801 individuals with IDD (509 males and 292 females) aged 18 to 43 (mean 33.1±8.5 years old) were recruited from the European Special Olympic Games in Spain 2009 and from the Spanish Special Olympic Games in 2010, 2011, 2012 [16]. After the screening, all participants were provided with specific information regarding their physical profile. Health education with an emphasis on musculoskeletal issues was also provided, using educational materials tailored for people with special educational reading levels when necessary. All of the participants had been diagnosed with mild IDD by a specialized doctor and their parent and/or guardian confirmed the diagnosis. All of the individuals appeared to be healthy, which was determined by their health history obtained from the participants and their parent and/or guardian. The hours per week in which the participants practiced physical activity or sports were recorded in order to classify the participants regarding their level of activeness [17]. Participants were identified as highly physically active (PA) when they practiced physical activities for three or more hours per week and as low PA when they practiced for less than three hours per week.

The exclusion criteria were: 1) any contraindications to exercise as assessed by a medical history questionnaire; 2) documented atherosclerotic heart disease; 3) documented atlantoaxial instability; 4) uncorrected congenital heart disease; and 5) an implanted pacemaker. The institutional review board at the University of Malaga approved the procedures used in this study, and all of the participants and their parents or guardians gave their written informed consent before participation. All participants were examined by a physiotherapist from the participating residency programs. All physiotherapists were trained in a four-hour session on how to interact with this specific population. The participants chose whether to have problem-focused examinations or more general examinations. All of the participants received counseling and education from the physical therapist and after the screening they were provided with musculoskeletal-specific patient education materials tailored for persons with lower reading levels.

### Procedures

The participants performed the following tests:

1. the *timed-stand test (TST)*, which requires the subject to complete 10 full stands from a seated position as quickly as possible without the use of their arms (measured in seconds) [18].
2. the *single leg stance test* with open and closed eyes (*SLST*), which requires the subject to stand on one leg with their eyes open or closed. They had to maintain their balance for as long as possible (in seconds). The arms were placed at the sides with the elbows slightly flexed during the test. The test continued until the subject lost balance, or put the other foot on the floor [19].
3. the *Functional Reach Test (FRT)*, which requires the subject to reach forward beyond the length of his/her arm without losing his balance. The participant was on two legs, positioned shoulder width apart (or seated if the athlete could not stand). The athlete was requested to lift one arm up to 90°, forward flexion and extend fingers. Test-retest reliability and validity was established in a previous study [20];
4. the *Expanded Timed-Get-up-and-Go Test (ETGUG)*, where all subjects used an armless chair and were instructed to not to use their arms to stand up. Although in traditional ETGUG an armchair is used [21], we used an armless chair. Previous studies explored the test using armless chairs [22] [23], that could reduce the variability between subjects by eliminating the choice to use or not to use the armrests to arise [24]. ETGUG test used a 10-meters walkway to include more gait cycles during the test [23]. Markings of the beginning and the end of the walkway were marked with 2.5 cm green tape on the floor. The tape markings were shown to the subjects before the trials. Subjects were instructed to sit straight and their backs touching the back of the chair. After they were given the go signal by the tester, they arose from the chair, walked at their faster walking speed but without running, turned around right or left after passing the green tape at the end of the way (*Turn_TUG*), returned back to the start chair, turned around and sat down. The tester timed their performance with a stop-watch. Partial time in transition of 2 meter before/after turn was recorded how turn into ETGUG variable.

### Data analysis

All of the variables were log transformed in order to approximate a normal distribution. Data were analyzed using principal components analysis (PCA) with Oblimin rotation and Kaiser normalization. To assess the suitability of the data for factor analysis, the Kaiser-Mayer-Olkin (KMO) measure of sampling adequacy was computed. KMO scores above 0.90 are considered excellent. Bartlett's test of sphericity was also applied to examine the extent to which the correlation matrices departed from orthogonality. The scores from PCA were recorded by regression method. The data were standardized with a mean equal to 0 and standard deviation equal to 1.

We examined the construct validity of our proposed two-factor model underlying balance for adults with IDD. The components were dynamic and static balance. The values of the variables were calculated using the mean values of the right and left sides of the variables (functional reach and the single leg stance with eyes open and closed). Analysis was performed with SPSS version 21 for Mac.

## Results

### Characteristics of the sample

This study included 801 adults with 508 males. Total sample has mean age of 34.3 years old and 29.2 body mass index. Table 1 shows the characteristics of the sample.

**Table 1:** Descriptive and clinical characteristics of study participants (n = 801).

Descriptive variables	
Gender (n° males,%)	508 (63.5%)
Age, years (Mean (SD)	34.3 (9.5)
Height, meters (Mean (SD)	1.62 (0.12)
Weight, kilograms) (Mean (SD)	73.1 (16.4)
Body Mass Index (Mean (SD)	29.2 (5.1)
Waist Perimeter, meters (Mean (SD)	95.1 (13.0)
Physical Activity/hours per week, n° (%)	
Lower 2 hours per week	424 (53.0%)
Higher 2 hour per week	377 (46.8%)

Clinical variables, Mean (SD)	
Single leg stand test_Open eyes (seconds)	13.6 (12.1)
Single leg stand test_Closed eyes (seconds)	4.7 (6.5)
Functional reach test (centimeter)	34.4 (10.5)
Time-stand test (seconds)	21.1 (10.7)
ETGUG (seconds)	20.0 (7.3)
Turn_TUG (seconds)	5.1 (2.17)

### Correlations

Table 2 shows the relationships between the variables included in the model.

**Table 2:** Correlation coefficients among the variables of balance included in the model.

	Functional reach test	Single leg stand test_Open eyes	Single leg stand test_Closed eyes	Turn_TUG	ETGUG
Single leg stand test_Open eyes	,404(**)				
Single leg stand test_Closed eyes	,321(**)	,622(**)			
Turn_TUG	-,401(**)	-,319(**)	-,276(*)		
ETGUG	-,374(**)	-,324(**)	-,273(*)	,832(**)	
Time-stand test	-,225(**)	-,264(**)	-,226(**)	,556(**)	,739(**)

*Correlation is significant at the 0.05 level (2-tailed).

**Correlation is significant at the 0.01 level (2-tailed).

#### Primary results

**Figure 1:**
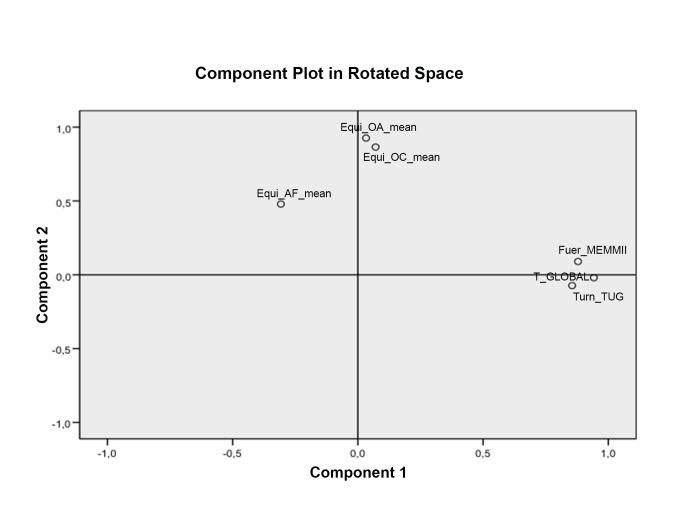
Component Plot and rotated space of the exploratory two- factor solution.

The KMO coefficient was 0.66, and Bartlett's test of sphericity was χ^2^ = 184.6, df = 15, *P*<0.001. In the PCA, the communalities ranged from 0.43 (functional reach) to 0.90 (Total time ETGUG). Two factors had eigenvalues greater than one and variance greater than 10%. The Component Plot and Rotated Space (see Figure 1) indicated that a two-factor solution (*Dynamic and Static Balance components*) was optimal. The PCA with direct Oblimin rotation revealed a satisfactory percentage of total variance explained by the two factors: 51.6 and 21.4%, respectively. The rotated component pattern matrix is shown in table 3.

**Table 3:** Factor loadings obtained in the principal component analysis (n = 801).

Pattern Matrix(a).	Component	
	Dynamic	Static
Functional reach test	-,308	**,479**
Single leg stand test_Open eyes	,032	**,926**
Single leg stand test_Closed eyes	,071	**,865**
Time-stand test	**,879**	,090
ETGUG	**,942**	-,020
Turn_TUG	**,855**	-,073

Extraction Method: Principal Component Analysis.

Rotation Method: Oblimin with Kaiser Normalization.

a Rotation converged in 5 iterations.

#### Median and Percentiles scores

The median score standardized for component dynamic of the balance index for adults with IDD was −0.181, and, the 20^th^, 40^th^, 60^th^ and 80^th^ percentiles were −0.832, −0.333, 0.017, and 0.908, respectively. The median score standardized for component static of the balance index for adults with IDD was −0.256, and the 20^th^, 40^th^, 60^th^ and 80^th^ percentiles were −0.640, −0.424, 0.118, and 0.285, respectively.

## Discussion

The present work attempted to capture the underlying components of balance in IDD by means of the multivariate statistical technique of factor analysis with the objective of using these dimensions to develop a new index of balance in IDD. To our knowledge, this is the first study to confirm that a two-factor model may be able to underpin a balance index for adults with IDD. In addition, we have developed a balance index for adults with IDD in order to assess balance using a single value in this population group. Our data reveal that a two-factor model underlies the balance construct for adults with IDD.

The two-factor solution that emerged in the PCA accounted for a significant proportion of variance, as well as preliminary evidence of construct validity. As we expected, the Dynamic component had a stronger relationship with the TST, ETGUG and Turn_TUG than with FRT and SLST, whereas the static dimension showed the opposite pattern. Secondly, our results indicate that a new Balance Index in adults with IDD can be identified pragmatically using a continuous index for sum of dynamic and static components based on the two-factor model obtained through PCA.

Falls constitute a special topic for adults with IDD, and there is a growing concern on research about falls management in people with IDD [25]. Studies indicate that the prevalence rate of falls among adults with IDD living in the community settings is substantially higher than in the general population. In several recent studies, the prevalence of falls among adults with IDD (20 to 80 years) ranged from 29% to 57% [25] [26] [27]. Among adults living in congregate care settings, the rate of falls was even higher: 70% of residents aged from 18 to 77 years fell in a 5-year period, and 79% of those resulted in injury [28]. Other studies have shown that people who have IDD, have a lower balance, both static and dynamic, than those without IDD [29] [30] [31]. Results from a recent study showed that even the functionally more able subjects of the sample had poor balance capacities, similar to adults in the general population aged around 20 years older [32]. Balance capacities decreased with increasing age and females had poorer balance capacities than males. Difficulties understanding the task and physical limitations were most often the reasons for drop-out while assessing balance [32]. Unlike the aging population without IDD, few standardized performance measures exist to assess physical function and risk for adverse outcomes such as nonfatal, unintentional injuries. Low balance capacities of older adults with IDD show the need for regular screening and the urge to facilitate a balance assessment in adults with IDD.

A key strength of the balance index is its' ability to combine existing measures into a single measure with superior validity, without increasing testing burden. Given the prevalence of balance problems in a population with IDD [7] and morbidity and mortality associated with falls and related injuries [33], a psychometrically strong and efficient balance measure that is a more sensitive indicator of fall risk would have important applications in IDD rehabilitation.

There is an urge for fall prevention programs for individuals with IDD. Studies have shown that supervised training in subjects with IDD improved physical performance in ADLs [34]. Appropriate physical activity [35] should play an important role for the health of individuals with IDD and diminish the impact of the subsequent onset of disability. Our results suggest that measures of balance may be valuable in identifying persons who are currently not disabled but are at increased risk for subsequent disability and therefore good candidates for a trial of an intervention to prevent disability.

One possible limitations could be the lack of social and cognitive factors in the model, as these could act as determinants of the functional performance of persons with IDD. Another possible weakness could be the relative homogeneity of the sample; it is unclear whether these results could be generalized to more diverse populations in terms of ethnicity, education and cultural country differences. Finally, it is possible that our results may not apply to adults from geographical areas other than those included in this study. The lack of a standard definition of fitness for adults with IDD is partly responsible for the disparity in the prevalence estimates reported to date.

In conclusion, our study may lead to improvements in the understanding and assessment of balance for adults with IDD. First, it confirms that a two-factor model may underlie the balance construct, and second, it provides an index that may be useful for identifying the balance level for adults with IDD.
